# From need to neglect: Exploring psychological barriers to preventive interventions in pregnancy

**DOI:** 10.1371/journal.pgph.0004826

**Published:** 2025-06-24

**Authors:** Dennis Bardoe, Robert Bagngmen Bio, Daniel Hayford, Denis Dekugmen Yar

**Affiliations:** 1 Department of Public Health Education, Akenten Appiah-Menka University of Skills Training and Entrepreneurial Development, Mampong, Ghana; 2 Department of Integrated Science Education, Akenten Appiah-Menka University of Skills Training and Entrepreneurial Development, Mampong, Ghana; PLOS: Public Library of Science, UNITED STATES OF AMERICA

## Abstract

To effectively reduce the impact of hepatitis B and malaria during pregnancy, consistent adherence to key preventive interventions such as vaccination, the use of long-lasting insecticidal nets (LLINs), and the administration of Sulfadoxine-Pyrimethamine (SP) is essential. Achieving high coverage of these interventions, however, requires a clear understanding of the factors that hinder compliance. Therefore, this study investigated the psychological determinants of non-compliance with these interventions among pregnant women in the Bono East Region. A prospective cross-sectional mixed-method study was conducted among 1,430 pregnant women. Data were collected through serological screening, questionnaires, in-depth interviews, and focus group discussions. Quantitative analysis included descriptive statistics, Chi-square tests, and logistic regression with results shown as crude and adjusted odds ratios at 95% confidence intervals. The quantitative analysis involved a four-step thematic analysis with a focus on transcription, profiling, coding, and thematic framework. The study revealed a series of psychological barriers to compliance. The fear of side effects (AOR = 2.17; 95% CI: 1.78 – 4.06), forgetfulness (AOR = 6.02; 95% CI: 2.13 – 9.91), and pain (AOR = 2.95; 95% CI: 1.97 – 4.94) increased the odds of hepatitis B infection. Likewise, fear of side effects (AOR = 2.07; 95% CI: 1.66 – 3.72), absence of symptoms (AOR = 1.82; 95% CI: 1.53 – 2.26), forgetfulness (AOR = 2.41; 95% CI: 1.48 – 3.90), perceived efficacy of herbs (AOR = 2.35; 95% CI: 1.49 – 3.68), pain (AOR = 2.52; 95% CI: 1.48 – 4.27), uncertainty (AOR = 2.38; 95% CI: 1.48 – 3.84), and prolonged adherence (AOR = 1.71; 95% CI: 1.10 – 2.66) increased the odds of malaria. The identified barriers and their strong association with increased odds of infections call for a groundbreaking and policy-driven public health response. Consequently, integrating psychosocial support and mental health services into antenatal care could play a crucial role in overcoming these barriers.

## Introduction

Public health in sub-Saharan Africa faces persistent threats from infectious diseases such as hepatitis B (HBV) and malaria [1] . Much attention has been paid to the recent outbreaks of viral hepatitis B (HBV) and malaria in sub-Saharan Africa [2,3]. This is because there is considerable evidence to support the fact that the outbreaks of these life-threatening diseases not only result in many fatalities in areas with highly recorded cases, but may also become a challenge that affects people’s health, wealth, and life outcomes. Unlike HBV, malaria is not transmissible from person to person but is transmitted through the bites of female *Anopheles* mosquitoes [4]. The epidemiological burden of malaria in Africa remains disproportionately high. As of 2021, the African region accounted for approximately 95% of global malaria cases and 96% of malaria-related deaths [5]. Within this context, an estimated 13.3 million pregnancies in sub-Saharan Africa were exposed to malaria, a scenario that could have led to about 961,000 low-birth-weight infants if no pregnancy-specific interventions had been implemented [6]. Because of the parasite species’ life cycle, which involves them developing, maturing, reproducing, and being discharged into the bloodstream to infect more erythrocytes and hepatocytes, malaria symptoms could be cyclic or periodic [7]. Common symptoms include fever, headache, nausea, and flu-like conditions, though symptom severity varies depending on the *Plasmodium* species involved and the individual's immune response [4]. Some studies on malaria in pregnancy have been conducted in various parts of Ghana, yielding evidence indicating intra- and inter-regional variations. Some of these included the prevalence of 20.4% in the Bono East Region [8] and 15.5% in the Northern Region [9].

Equally important is the public health threat posed by hepatitis B virus (HBV), especially among pregnant women. HBV primarily infects liver cells, and its clinical manifestations result from interactions between the virus and the host's immune system. If untreated, this could progress to cirrhosis and hepatocellular carcinoma [10]. Approximately 33% of the world’s population has a serological confirmation of current or previous HBV infection, of which only 10% of chronic cases have been diagnosed [10]. Most of the people diagnosed and receiving treatment live in the Americas, Europe, Asia, or the Western Pacific [11]. In contrast, Africa lags behind, with less than 2% of cases diagnosed and only 0.1% receiving treatment, , resulting in at least 200,000 deaths each year [11]. In Ghana, studies on HBV infection among pregnant women have been conducted in different parts of the country with varying rates of occurrence. Some of these include a prevalence of 12.6% in the Kumasi Ashanti Region [12] and 3.3% in the Ningo-Prampram District, Greater Accra [13].

These trends highlight the urgent need for effective interventions to protect maternal and child health. In response, a range of targeted strategies and comprehensive guidelines have been introduced. These efforts include strengthening disease surveillance systems, expanding vaccination coverage, and managing chronic conditions more effectively. In addition, public awareness campaigns have been launched, alongside the administration of immunoprophylaxis. Likewise, malaria preventive strategies such as the distribution of long-lasting insecticidal nets (LLINs), increased use of Intermittent Preventive Treatment with Sulfadoxine-Pyrimethamine (IPTp-SP) among pregnant women, and the implementation of indoor residual spraying (IRS) have also been prioritized [14,15]. However, the success of these public health interventions ultimately hinges on individual compliance. Promoting adherence requires a clear understanding of the factors that may hinder it. Gaining such insights could inform the development of targeted strategies and foster a more holistic approach, ensuring that interventions are not only accessible but also effectively implemented. This, in turn, could significantly reduce the burden of infectious diseases in the Bono East Region and beyond. Despite these efforts, there remains a critical gap in research focused on behavioural determinants of non-compliance. To the best of our knowledge, no peer-reviewed studies have specifically examined the psychological factors influencing non-compliance with integrated interventions among pregnant women in this region. Most existing research has centered on epidemiological trends and intervention coverage, leaving an important gap in understanding adherence behaviour. This knowledge gap served as the rationale for the present study, which aimed not only to contribute to academic literature but also to provide actionable insights into barriers that may exacerbate the severity and impact of these diseases.. In light of these gaps, this study investigated the psychological determinants of non-compliance with HBV vaccines, LLINs, and IPTp-SP among pregnant women in the Bono East Region of Ghana. By identifying these determinants, the findings of this study could significantly unearth more effective and groundbreaking strategies to enhance compliance and improve maternal and child health outcomes. The study was grounded on the question: “*What psychological determinants compromise compliance with HBV and malaria interventions (LLINs, IPTp-SP, and HBV vaccines) among pregnant women?”*

## Materials and methods

### Ethical approval

The study and all associated protocols were reviewed and approved by the Committee on Human Research, Publication, and Ethics (CHRPE), Kwame Nkrumah University of Science and Technology, School of Medical Sciences, under the Declaration of Helsinki (assigned approval number: CHRPE/AP/1081/23). Participants signed or thumbprint a written consent form after receiving a thorough explanation before engaging in the study. Consent was also sought from the parents or guardians of participants aged below 18 years.

### Study setting

**Fig 1:**
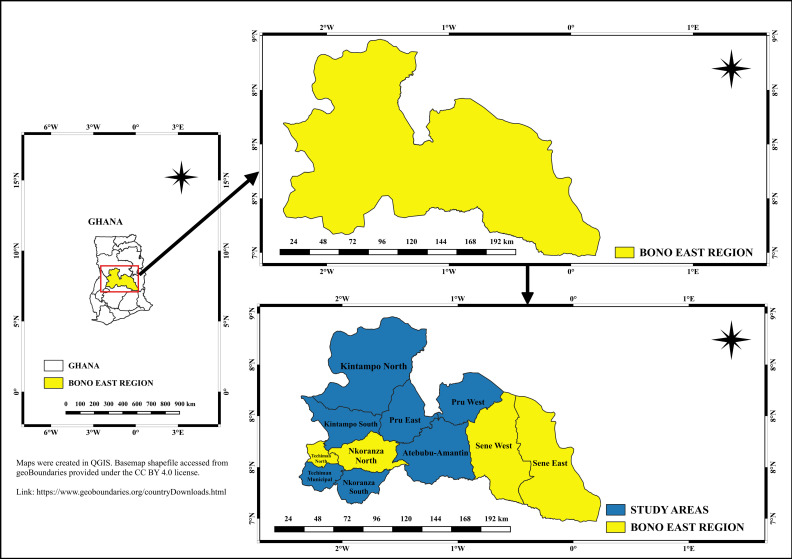
Study Sites. Maps were created in QGIS. Basemap shapefile accessed from geoBoundaries provided under the CC BY 4.0 license [17]. The shapefiles are publicly accessible. https://www.geoboundaries.org/countryDownloads.html.

The study was conducted in the Bono East Region of Ghana. The region is made up of approximately 1,203,400 people, occupying 22,952km^2^ piece of land, with a population density of 48.75 per sq km [16]. The study included seven health facilities in seven municipalities/districts: Atebubu-Amantin Municipality, Kintampo South District, Kintampo North Municipality, Nkoranza South Municipality, Techiman Municipality, Pru East Municipality, and Pru West District, as shown in **Fig 1**. The selection of health facilities for this study was based on strategic considerations to ensure comprehensive and representative data collection. Thus, these facilities were selected because they serve as primary points of healthcare delivery for pregnant women and provide antenatal care (ANC) services. Their strategic locations allow access to a diverse population, encompassing both rural and urban settings, which is crucial for capturing the varied determinants of non-compliance and their association with the prevalence of HBV and malaria.

### Study design

A prospective hospital-based cross-sectional mixed-method study was conducted among 1,430 pregnant women in seven selected municipality/district health facilities from 22^nd^ January to 15^th^ April 2024. It is part of a broader study on the epidemiology of comorbidities among pregnant women in the Bono East Region.

### Study population

The study comprised consented pregnant women who attended ANC in selected healthcare facilities.

### Inclusion

Consented pregnant women who dwelled in the selected municipalities/districts and attended ANC at the selected health facilities were included in the study.

### Exclusion criteria

Pregnant women who felt uncomfortable during the recruitment process and those who did not give consent due to personal reasons were excluded. In rare cases where no interpreter was available for a participant’s dialect, the participant was respectfully excluded.

### Sample size estimation

The sample size was determined using Slovin’s formula. After determining the sample size of 1075, 35% of 1075, which was approximately 377, was added to give a total sample size of 1452. The rationale was to ensure sufficient statistical power and reliability from the sample for analysis and to compensate for non-response [18,19]. In addition, it was also to satisfy one of the assumptions of the logistic regression analysis, which emphasises the presence of a larger sample size [20]. Afterwards, each study site was allotted a quota based on the sample size proportional to the respective municipality/districts to maintain representativeness. Pregnant women were randomly selected until their numbers were proportional to the sample size of the representative municipalities/districts, as shown in Table 1.

**Table 1:** Proportional allocation of sample size across each selected municipality/district.

Municipality/District	The population of pregnant women recorded in 2022	Sample proportion [%]	Estimated sample size	Approximated sample size
Atebubu-Amantin Municipality	5192	15.6	226.5	226
Kintampo North Municipality	6186	18.5	268.6	269
Kintampo South District	2205	6.6	95.8	96
Nkoranza South Municipality	3932	11.8	171.3	171
Techiman Municipality	9334	27.9	405.1	405
Pru East Municipality	4140	12.4	180.1	180
Pru West District	2406	7.2	104.5	105
**Total**	**33395**	**100**	**1451.9**	**1452**

### Enrolment of participants

Pregnant women were enrolled from 13^th^ December 2023–18^th^ January 2024. After an initial examination to determine eligibility, those who consented were approached to discuss the study’s objectives. Twenty-two did not participate for various reasons, including feeling uncomfortable, a language barrier, a lack of time, and personal reasons, indicating a non-response rate of 1.52%.

### Variables of interest

#### Outcome variable.

**HBV and malaria mono-infections**. The outcome variables explored the status of HBV and malaria among pregnant women at the time of data collection. These variables were categorical and, measured on a dichotomous scale. They were coded into ("1 - Positive" and "2 - Negative").

#### Predictor variable.

The predictor variable for this study was a series of psychological barriers to compliance with interventions (including fear of side effects, absence of signs and symptoms, forgetfulness, perceived efficacy of traditional herbal medicine, a perception that formal care does not meet expectations, pain, uncertainty, misplaced trust in healthcare providers, distress about the death of a family member, and fatigue due to prolonged adherence). These predictor variables were all categorical and were coded into two categories (“0 – No” and “1 – Yes”).

#### Data collection methods.

Maternal (Obstetrics) parameters including gravidity, parity, ANC visit, trimester of pregnancy, Glucose-6-phosphate Dehydrogenase (G6PD) status, blood group, haemoglobin (Hb) levels, and sickling status were extracted from the ANC records book (using S2 Text. Clinical Data Collection Tool). These tests are routinely conducted as part of the standard ANC protocol in Ghana to ensure the early detection and management of complications during pregnancy [21]. In addition, a structured closed-ended questionnaire (S1 Text. Questionnaire) was used to collect data, particularly on the sociodemographic characteristics and barriers to compliance. The questionnaire was adapted from the Ghana Demographic and Health Survey (GDHS) and the Ghana Living Standards Survey Round 6 (GLSS6), which were modified to suit the purpose of this study. The modified questionnaire was presented to experts in quantitative and epidemiological studies to ensure content validity. Moreover, the questionnaire was pre-tested at Agyenkwa Hospital, Jema, which enabled the identification of ambiguous questions for correction before actual data collection.

Likewise, focus group discussions (FGDs) with consented pregnant women (using S3 Text. Focus Group Discussion Guide) and in-depth interviews (IDIs) with the consented health workers (using S4 Text. In-depth Interview Guide) were conducted to gather detailed qualitative information, particularly on preventive interventions and, barriers to adherence, to complement the qualitative data obtained. Participants were purposively selected based on their experiences with maternal healthcare services. This included pregnant women who had attended antenatal care (ANC) and senior midwives who had provided maternal care services within the selected health facilities. The selection also aimed to form groups with similar ethnic and linguistic backgrounds to foster a comfortable environment for open and honest discussion. With participants’ consent, all discussions were audio-recorded to support accurate data transcription and translation.

### Biases and mitigation strategies

In this study, several potential biases were identified and addressed to enhance the validity and reliability of findings. Firstly, sampling bias could occur due to the exclusion of individuals based on language barriers and differences between the populations of the selected municipalities and districts under study. This was addressed by the incorporation of multilingual support where feasible and by allotting a quota based on the sample size proportional to the respective municipality/districts to maintain representativeness. Secondly, selection bias was inherent due to the exclusion of pregnant women who did not attend ANC at the selected health facilities. This was minimised by ensuring broader accessibility by adding 377 pregnant women to the estimated 1075 and also randomisation, where every pregnant woman who met the eligibility criteria was given an equal chance of being selected. In turn, this ensured a representative distribution across relevant demographic and contextual variables. In addition, pregnant women may provide responses they believed were socially acceptable or favourable rather than truthful answers. This could lead to the overreporting of healthy behaviours, which could lead to response bias. To address this, pregnant women were assured of anonymity and confidentiality to encourage honest responses.

## Data management and statistical analysis

### Quantitative analyses

All data (S1 Data. Dataset) were examined for completeness, consistency, and clarity as part of data management before, analyses with STATA 14 (StataCorp, College Station, USA). Descriptive statistical analyses were performed to provide summary output of the frequency, percentage distribution, and mean with standard deviations (S.D.) for continuous variables. Similarly, Pearson’s chi-square tests were also performed to determine the differences in proportions. Moreover, logistic regression analysis was performed to identify the barriers significantly associated with HBV and malaria. To avoid prematurely excluding potential confounders and effect modifiers, all variables with a p-value ≤ 0.25 in the bivariate logistic regression (Model I) were included in the multivariate logistic regression (Model II). The results from these analyses are reported as crude odds ratios (COR) and adjusted odds ratios (AOR), with statistical significance determined at 0.05 (95% confidence interval).

### Logistic regression model

In this study, all measurements were observed, and there were no missing values. The general model of the logistic regression equation is expressed as;


$$\mathrm{\backslash [}log\;(p)=\;\ln\left(\frac{p}{1-p}\right)=\beta_{0}+\beta_{1}X_{1}+\beta_{2}X_{2}+\ldots +\beta_{k}X_{k}\mathrm{\backslash ]}$$

Where p is the probability of the outcome variables (HBV and malaria) occurring, $\beta_{0}$ is the intercept, and $\beta_{1},\beta_{2},\ldots \beta_{k}$ are the coefficients for the independent variables $X_{1},\;X_{2},\ldots X_{k}$.

### Qualitative analyses

Data-driven inductive thematic analysis was used to analyse qualitative data, with a focus on four steps: transcription, profiling, coding, and thematic framework. FGDs and IDIs with consented pregnant women and health workers were recorded and transcribed verbatim from the preferred language of participants into English. Identified data were profiled with background data using different variables. The validated transcripts were assigned identification codes according to the study location. The FGDs were labelled FGD-ATBM (Focus Group Discussion for Atebubu-Amantin Municipal), FGD-KSD (Focus Group Discussion for Kintampo South District), FGD-KNM (Focus Group Discussion for Kintampo North Municipal), FGD-NSM (Focus Group Discussion for Nkoranza South Municipal), FGD-TM (Focus Group Discussion for Techiman Municipal), FGD-PWD (Focus Group Discussion for Pru West District), and FGD-PED (Focus Group Discussion for Pru East District). Likewise, the IDIs were labelled IDI-ATBM (In-depth Interview for Atebubu-Amantin Municipal), IDI-KSD (In-depth Interview for Kintampo South District), IDI-KNM (In-depth Interview for Kintampo North Municipal), IDI-NSM (In-depth Interview for Nkoranza South Municipal), IDI-TM (In-depth Interview for Techiman South Municipal), IDI-PWD (In-depth Interview for Pru West District), and IDI-PED (In-depth Interview for Pru East District).

After profiling the transcripts, manual coding was conducted by identifying keywords in the interviews relevant to the study. Significant sections of the transcripts, based on the study’s objectives, were highlighted and assigned concise “labels” or, “codes” to summarise their content. For example: “Concern about the side effects of the intervention” was coded as “***Fear of side effects***.”, “The absence of signs or symptoms of HBV and malaria” became “***Absence of signs or symptoms***.”, “Often forget due to daily routine” was simplified to “***Forgetfulness***.”, “Perceived efficacy of traditional herbal medicine” was labelled “***Perceived efficacy of herbs***.”, “The perception that formal care does not meet expectations” was coded as “***Formal care***.”, “Feel pain to intervention” became “***Pain.***”, “Unsure of the necessity of intervention” was coded as “***Uncertainty***.”, “Lack of trust in health service providers” was labelled as “***Mistrust***.”, “Distress about the death of a family member” was labelled “***Distress***.” And, “Getting tired of the prolonged period of adherence” was coded as “***Prolonged adherence***.”

Microsoft Word was used to organise, edit, and categorise the data into themes aligned with the study’s objectives. Emerging patterns related to barriers to HBV vaccination adherence, use of LLINs, and uptake of IPTp-SP were identified. These themes were cross-checked against the original data to ensure consistency and accuracy. To strengthen the findings, illustrative quotes from participants were incorporated into the results, complementing the quantitative data.

### Theoretical foundation of the study

**Fig 2:**
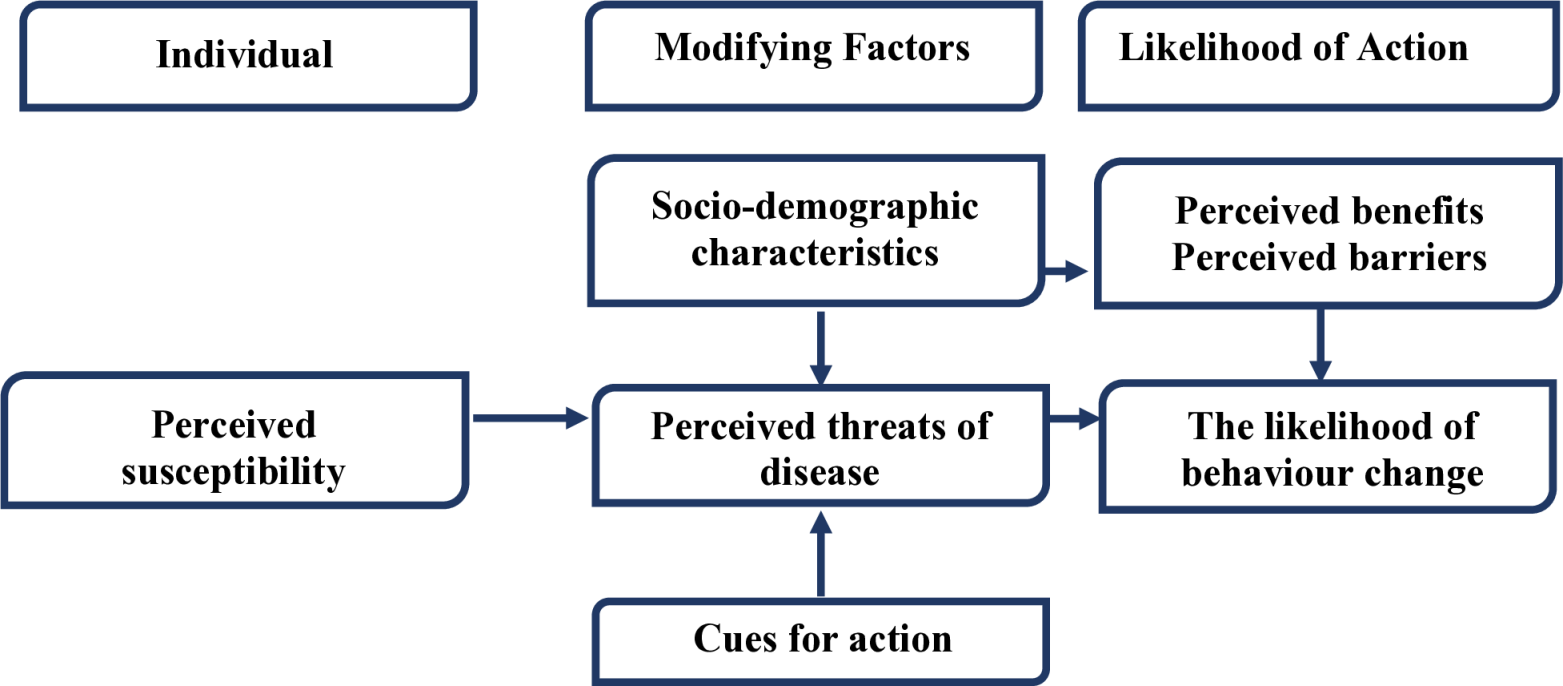
Theoretical Framework. [Adopted from Rosenstock et al. (1994] [23]].

This study was grounded in the Health Belief Model (HBM). It assumes that an individual’s course of action is frequently determined by their perceptions of the benefits and barriers associated with health behaviour [22]. The HBM has some primary indicators used to predict why people decide or do not decide, to control, prevent, or screen for different illness conditions [22]. These primary indicators include perceived susceptibility, perceived severity, perceived benefits, perceived barriers, cues for action, and self-efficacy [22]., as presented in **Fig 2**. The Health Belief Model (HBM) provides a theoretical lens to understand and address the psychological determinants influencing adherence to preventive health interventions for HBV and malaria among pregnant women.

## Results

### Sociodemographic and maternal characteristics of pregnant women

The sociodemographic and maternal characteristics of the pregnant women are presented in **Table 2**. Their ages at enrolment ranged from 17 to 40 years, with a mean of 28.8 ± 3.73 (95% C.I: 28.63 – 29.02). A large percentage (76.1%) were between the ages of 26 and 40 years. Most (76.1%) were married and 42.8% had attended primary school. Furthermore, 83.5% were Christians. Also, 97.6% were employed. They were engaged in various occupations, including hairdressing (19.2%), seamstress (16.4%), farming (14.3%), trading (42.9%), civil service (1.8%), and domestic activities (3.1%). Most pregnant women (48.4%) earned between Ghç100 and 500. A large majority (75.9%) lived in extended households. In addition, 70.6% lived in compound houses. The majority (89.1%) lived in houses made of blocks with iron sheets. A substantial majority (62.6%) lived in households whose capacity was 1–5.

**Table 2:** Sociodemographic and obstetric characteristics of pregnant women.

Variable	Categories	[n]	[%]
**Age**	<18 years	7	0.5
	18-25	167	11.7
	26-30	1088	76.1
	31-40	168	11.7
**Marital status**	Not married	118	8.2
	Married	1308	91.5
	Cohabitation	4	0.3
**Educational attainment**	No formal education	414	29
	Primary education	612	42.7
	Junior High School	251	17.6
	Senior High School	127	8.9
	Tertiary	26	1.8
**Religious affiliation**	Islam	233	16.3
	Christianity	1194	83.5
	African tradition	3	0.2
**Labour force**	Employed	1396	97.6
	Unemployed	34	2.4
**Type of occupation**	Hairdressing	275	19.2
	Seamstress	234	16.4
	Farming	204	14.3
	Civil service	26	1.8
	Trading/Marketing	613	42.8
	Domestic activities	44	3.1
	Housewife (unemployed)	14	1.0
	Student (unemployed)	6	0.4
	None (unemployed)	14	1.0
**Monthly income**	None	34	2.4
	Ghç 100–500	692	48.4
	Ghç 600–1000	538	37.6
	Above Ghç 1100	166	11.6
**Household structure**	Extended	1086	75.9
	Nuclear	344	24.1
**Household type**	Compound House	1009	70.6
	Self-Contain House	421	29.4
**Household category**	Mud with thatch	27	1.9
	Mud with iron sheets	129	9.0
	Blocks with iron sheets	1274	89.1
**Number of people in a household**	1-5	895	62.6
	6-10	532	37.2
	11-15	3	0.2
**Gravidity**	Primigravida	335	23.4
	Secundigravida	624	43.6
	Multigravida	471	33.0
**Parity**	Nulliparous	296	20.7
	Multiparous	1134	79.3
**ANC Visits**	1-3	990	69.3
	4-6	340	23.7
	7-8	100	7.0
**Trimester**	First trimester	1051	73.5
	Second trimester	256	17.9
	Third trimester	123	8.6
**G6PD**	No defect	1371	95.9
	Partial defect	49	3.4
	Full defect	10	0.7
**Blood Group**	A	422	29.5
	B	420	29.4
	AB	251	17.6
	O	337	23.6
**Sickling**	Positive	45	3.1
	Negative	1385	96.9

n, Frequency; %, Percentage

In line with gravidity, a majority (43.6%) were secundigravida. Also, 79.3% were multiparous. Antenatal care (ANC) visits ranged from 1 to 9, with a mean attendance of 2.85 ± 1.9 (95% CI: 2.76 – 2.95). The ANC visits for the majority of the pregnant women (69.3%) ranged between 1 and 3 times. Also, 73.5% were in their first trimester. Regarding G6PD status, 95.9% had no defect. Finally, a significant proportion (29.5%) had blood type A. The haemoglobin (Hb) levels ranged from 6.2 to 13.3 g/dl, with a mean Hb of 9.76 ± 1.09 g/dl (95% CI: 9.71 – 9.82). A majority (59%) had moderate anaemia. A substantial majority (96.9%) had no sickle cell trait, as shown in **Table 2**.

### Prevalence of HBV and malaria mono-infection among pregnant women

As part of the study, the prevalence of HBV and malaria among consented pregnant women was assessed. The prevalence of HBV was 1.8% (95% CI: 1.24 – 2.65) [19], whereas malaria was 10.8% (95% CI: 9.32 – 12.56) [18], as presented in **Table 3**.

**Table 3:** Prevalence of HBV and malaria mono-infection among pregnant women.

Data Collection Site	Participant’s HBV status				Participant’s malaria status			
	Positive		Negative		Positive		Negative	
	[n]	[%]	[n]	[%]	[n]	[%]	[n]	[%]
Atebubu-Amantin Municipality	3	0.2	220	15.4	19	1.3	204	14.3
Kintampo North Municipality	4	0.3	261	18.3	27	1.9	238	16.6
Kintampo South District	1	0.1	93	6.5	9	0.6	85	5.9
Nkoranza South Municipality	5	0.3	164	11.5	18	1.3	151	10.6
Techiman Municipality	7	0.5	392	27.4	43	3	356	24.9
Pru East Municipality	4	0.3	173	12.1	28	2	149	10.4
Pru West District	2	0.1	101	7.1	11	0.8	92	6.4
**Total**	**26**	**1.8**	**1404**	**98.3**	**155**	**10.8**	**1275**	**89.2**

n, Frequency; %, Percentage

### Availability of interventions for collective control of HBV and malaria in pregnancy

From in-depth interviews, a series of interventions and preventive guidelines were available to ensure a collective control of HBV and malaria among pregnant women. These included regular screening for HBV and malaria, HBV vaccination, distribution and promotion of long-lasting insecticidal nets (LLINs), administration of antimalarial drugs such as intermittent preventive treatment in pregnancy with Sulfadoxine-Pyrimethamine (IPTp-SP), as well as providing information and education on preventive measures. These were remarked upon by the following quote:

*“... For malaria prevention, we provide pregnant women with ITNs, ensuring they understand how to use them properly and emphasising the importance of maintaining a clean environment to prevent mosquito breeding. In addition, we administer Intermittent Preventive Treatment in Pregnancy with Sulfadoxine-Pyrimethamine (IPTp-SP) to reduce the risk of malaria infection. Regarding hepatitis B virus (HBV), we screen all pregnant women who visit our facility. Those who test negative are counselled on their status and educated on ways to protect themselves from HBV transmission. For those who test positive, we provide counselling and strongly encourage them to receive immunoglobulin treatment, which can be costly at around GH¢ 900, to prevent transmission to their infants. Since HBV is not curable, we focus on helping them manage the condition effectively...” – (In-charge, IDI-PWD)*

### HBV vaccination coverage, utilization of LLINs and uptake of IPTp-SP among pregnant women

The study assessed the HBV vaccination coverage, utilisation of LLINs, and uptake of IPTp-SP among the pregnant women studied. As presented in **Table 4**, the majority of the pregnant women, 74.4% (95% CI: 72.1 – 76.6) [19], have not been vaccinated against HBV. Similarly, a significantly large proportion of the pregnant women, 60.3% (95% CI: 57.8 – 62.9) [19], do not use the LLINs provided to them during their routine ANC visits. Of the pregnant women studied, 73.5% were in their first trimester and hence were not due for IPTp-SP. Among the remaining (26.5%), 17.9% and 8.6% were in their second and third trimesters, respectively. Among the pregnant women due for IPTp-SP, 62.8% (n = 238), 21.6% (n = 82), 9.0% (24) and 6.6% (25) had received ≤1, 2, 3 or ≥4 doses of IPTp-SP, respectively [19].

**Table 4:** HBV vaccination coverage, utilisation of LLINs and uptake of IPTp-SP among pregnant women.

	HBV vaccination coverage				
	Vaccinated		Not vaccinated		Total
**Data Collection Site (Health Facility)**	**[n]**	**[%]**	**[n]**	**[%]**	**[n]**
Atebubu-Amantin Municipality	79	5.5	144	10.1	223
Kintampo North Municipality	64	4.5	201	14.1	265
Kintampo South District	12	0.8	82	5.7	94
Nkoranza South Municipality	23	1.6	146	10.2	169
Techiman Municipality	145	10.1	254	17.8	399
Pru East Municipality	30	2.1	147	10.3	177
Pru West District	14	1	89	6.2	103
**Total**	**367**	**25.6**	**1063**	**74.4**	**1430**
	**LLINs utilisation**				
	**Yes**		**No**		**Total**
**Data Collection Site (Health Facility)**	**[n]**	**[%]**	**[n]**	**[%]**	**[n]**
Atebubu-Amantin Municipality	55	3.8	168	11.7	238.5
Kintampo North Municipality	119	8.3	146	10.2	283.5
Kintampo South District	29	2	65	4.5	100.5
Nkoranza South Municipality	31	2.2	138	9.7	180.9
Techiman Municipality	166	11.6	233	16.3	426.9
Pru East Municipality	88	6.2	89	6.2	189.4
Pru West District	79	5.5	24	1.7	110.2
**Total**	**567**	**39.7**	**863**	**60.3**	**1530**
	**Uptake of IPTp-SP**				
	**Doses**				
**Data Collection Site (Health Facility)**	**≤1**	**2**	**3**	**≥4**	**Total**
Atebubu-Amantin Municipality	27	13	4	2	46
Kintampo North Municipality	33	10	6	9	58
Kintampo South District	16	9	2	5	32
Nkoranza South Municipality	37	16	7	2	62
Techiman Municipality	72	20	9	4	105
Pru East Municipality	31	9	4	2	46
Pru West District	22	5	2	1	30
**Total**	238	82	34	25	379
**%**	**62.8**	**21.6**	**9.0**	**6.6**	**100**

n, Frequency; %, Percentage

### Psychological determinants of non-compliance with HBV vaccines, LLINs, and IPTp-SP

Various psychological barriers to adherence to HBV and malaria interventions (LLINs, IPTp-SP, and HBV vaccines) were assessed. These included fear of side effects, absence of signs and symptoms, forgetfulness, perceived efficacy of traditional herbal medicine, a perception that formal care does not meet expectations, pain, uncertainty, misplaced trust in healthcare providers, distress about the death of a family member, and fatigue due to prolonged adherence. Referring to **Table 5**, a considerable number of pregnant women (25%) cited concerns about the side effects of the interventions as a reason for their non-adherence. Similarly, 41.9% emphasised the absence of signs and symptoms of HBV and malaria as a reason for not adhering to the interventions. In addition, a smaller proportion of pregnant women (16.6%) identified forgetfulness as a barrier to adherence. Furthermore, a substantial minority of the studied pregnant women (23.2%) indicated a preference for herbal medicine because of its perceived efficacy as a reason for non-adherence to the interventions.

**Table 5:** Psychological barriers to adherence to intervention.

Theme (Barriers)	Code	Response			
		Yes		No	
		[n]	[%]	[n]	[%]
Concern about the side effects of the intervention	Fear of side effects	358	25.0	1072	75.0
The absence of signs or symptoms of HBV and malaria	Absence of signs or symptoms	599	41.9	831	58.1
Often forget due to daily routine	Forgetfulness	237	16.6	1193	83.4
Perceived efficacy of traditional herbal medicine	Perceived efficacy of herbs	332	23.2	1098	76.8
The perception that formal care does not meet expectations	Unmet expectations of care	106	7.4	1324	92.6
Feel pain to intervention	Pain	189	13.2	1241	86.8
Unsure of the necessity of intervention	Uncertainty	386	27.0	1044	73.0
Lack of trust in health service providers	Mistrust	258	18.0	1172	82.0
Distress about the death of a family member	Distress	169	11.8	1261	88.2
Getting tired of the prolonged period of adherence	Prolonged period of adherence	460	32.2	970	67.8

n, Frequency; %, Percentage

A small proportion of the studied pregnant women (7.4%) perceived that formal care did not meet their expectations, which in turn was a barrier to adhering to the interventions provided to them. Likewise, some pregnant women (13.2%) identified pain as a barrier to adherence to interventions. Furthermore, a significant minority of pregnant women (27%) cited uncertainty as a psychological barrier to adherence. These women expressed uncertainty about the necessity of the interventions. Moreover, some of the pregnant women (18%) explained that a lack of trust in healthcare providers was the reason they failed to adhere to the interventions. Subsequently, a small but significant proportion of pregnant women (11.8%) mentioned distress over the death of a family member because of adherence as a psychological barrier. Lastly, a notable proportion of the studied pregnant women (32.2%) reported feeling tired due to the prolonged period of adherence to interventions as a barrier to adhering to HBV and malaria interventions.

Qualitatively, the FGDs with selected pregnant women yielded valuable insights, as participants provided remarks that supported the identified psychological barriers.

Regarding the fear of side effects, the participants made the following remarks:

*“... After the first injection (HBV vaccine), I could not raise my arm for nearly 3 days. Later, the injection site became infected and developed a sore. It was only after seeking help from Omaala (herbal practitioner) that the sore healed...” – (5*^*th*^ *Pregnant Woman, FGD-TM)*

*“… When I took the drug (IPTp-SP), I vomited and became very weak…” – (10*^*th*^ *Pregnant Woman, FGD-KNM)*

Regarding the absence of signs or symptoms, another pregnant woman emphasised:

*“... I do not notice the presence of mosquitoes. I rarely experience headaches, high body temperature, or joint pains. I only feel joint pains when I go to the farm. So, if these are the signs that indicate I should sleep under a mosquito net, then I’m sorry...” – (3*^*rd*^ *Pregnant Woman, FGD-NSM)*

### Regarding forgetfulness, a pregnant woman commented

*“... When the nurses advised me to get the injection (HBV vaccine), I followed through and was informed that I needed three injections for complete vaccination. I took two but forgot the exact date for the third. Last month, when they told us to get the injection again, the person there said the two I had taken were wasted, and I needed to start over...” – (4*^*th*^ *Pregnant Woman, FGD-PED)*

In line with the perceived efficacy of herbal medicine, a pregnant woman commented:

*“... Every time I take the IPTp-SP medication, I feel weak, which prevents me from selling my food. That is why I do not come here (ANC). Instead, if I am feeling unwell, I go to Kwapongs pharmacy to get Taabea Herbal...” – (6th Pregnant Woman, FGD-ATM)*

Regarding the lack of trust in health service providers, a pregnant woman highlighted the following:

*“... Some nurses here do not take the time to explain the importance of the injection. If you ask them questions, they will respond rudely. I will not take the injection if I do not understand its purpose...” – (3*^*rd*^ *Pregnant Woman, FGD-PED)*

Regarding distress about the death of a family member, a pregnant woman commented:

*“... Two years ago, some health workers came to educate us about HBV at the community centre. After the session, they advised us to get the injection at the nearest health facility. At that time, I did not have the money to go. However, my sister (Monica) went and received the injection. Within a week, she started getting sick, which eventually led to her death. Later, we learned that she had developed a boil in her ear, and the injection intensified the condition, resulting in her death...” – (10*^*th*^ *Pregnant Woman, FGD-TM)*

Regarding becoming tired of the prolonged period of adherence, a pregnant woman mentioned the following:

*“… As I mentioned earlier, the SP medication they provide during ANC visits is quite large, making it challenging to swallow. If you manage to swallow it, you often feel like vomiting afterwards. Imagine having to take this medication regularly until you are close to giving birth. It’s quite alarming to me…” – (7*^*th*^ *Pregnant Woman, FGD-KNM)*.

**Table 6** presents the association between various aspects of psychological barriers and HBV. In the bivariate analysis, fear of side effects, absence of signs and symptoms, forgetfulness, perceived efficacy of herbal medicine, pain, uncertainty, and fatigue due to prolonged adherence were significantly associated with HBV mono-infection. In the multivariate analysis, only fear of side effects, forgetfulness, pain, and distress about the death of a family member were independent barriers significantly associated with HBV mono-infection. Specifically, pregnant women who did not adhere to HBV vaccination due to fear of side effects were 2.17 times more likely to be infected with HBV compared with those who adhered (AOR = 2.17; 95% CI: 1.78 – 4.06). Similarly, those who identified forgetfulness as a barrier to adherence had extremely higher odds of being infected with HBV (AOR = 6.02; 95% CI: 2.13 – 9.91). Furthermore, pregnant women who highlighted pain as a barrier to adherence had approximately three times the odds of being infected with HBV (AOR = 2.95; 95% CI: 1.97 – 4.94). Similarly, the odds of HBV were approximately five times higher among pregnant women, indicating distress about the death of a family member as a barrier to compliance (AOR = 4.98; 95% CI: 1.72 – 6.46)

**Table 6:** Association between psychological barriers and HBV mono-infection among pregnant women.

		HBV status							
	**Frequency**	**Positive**	**Negative**	Model I			Model II		
**Variable**	**n [%]**	**n [%]**	**n [%]**	**COR**	**[95%CI]**	**p-value**	**AOR**	**[95%CI]**	**p-value**
**Fear of side effects**									
Yes	358 [25.0]	16 [1.1]	342 [23.9]	4.96	[2.23 - 6.05]	0.000^*^	2.17	[1.78 - 4.06]	0.017^*^
No	1072 [75.0]	10 [0.7]	1062 [74.3]	1			1		
**Absence of signs or symptoms**									
Yes	599 [41.9]	18 [1.3]	581 [40.6]	3.18	[1.37 - 5.37]	0.007^*^	2.09	[0.73 - 5.97]	0.166
No	831 [58.1]	8 [0.6]	823 [57.6]	1			1		
**Forgetfulness**									
Yes	237 [16.6]	16 [1.1]	221 [15.5]	8.56	[3.83 - 12.11]	0.000^*^	6.0	[2.13 - 9.91]	0.001^*^
No	1193 [83.4]	10 [0.7]	1183 [82.7]	1			1		
**Perceived efficacy of herbs**									
Yes	332 [23.2]	11 [0.8]	321 [22.4]	2.47	[1.12 - 5.44]	0.024^*^	1.32	[0.49 - 3.54]	0.172
No	1098 [76.8]	15 [1.0]	1083 [75.8]	1			1		
**Unmet expectations of care**									
Yes	106 [7.4]	3 [0.2]	103 [7.2]	1.64	[0.48 - 5.57]	0.122	0.29	[0.06 - 1.30]	0.107
No	1324 [92.6]	23 [1.6]	1301 [91.0]	1			1		
**Pain**									
Yes	189 [13.2]	14 [1.0]	175 [12.2]	8.19	[3.72 - 11.04]	0.000^*^	2.95	[1.97 - 4.94]	0.035^*^
No	1241 [86.8]	12 [0.8]	1229 [85.9]	1			1		
**Uncertainty**									
Yes	386 [27.0]	12 [0.8]	374 [26.2]	2.36	[1.08 - 5.14]	0.031^*^	0.37	[0.11 - 1.23]	0.108
No	1044 [73.0]	14 [1.0]	1030 [72.0]	1			1		
**Mistrust**									
Yes	258 [18.0]	3 [0.2]	255 [17.8]	0.58	[0.17 - 1.97]	0.190	0.15	[0.03 - 0.73]	0.058
No	1172 [82.0]	23 [1.6]	1149 [80.3]	1			1		
**Distress**									
Yes	169 [11.8]	8 [0.6]	161 [11.3]	3.43	[1.46 - 5.01]	0.004^*^	4.98	[1.72 - 6.46]	0.003^*^
No	1261 [88.2]	18 [1.3]	1243 [86.9]	1			1		
**Prolonged period of adherence**									
Yes	460 [32.2]	16 [1.1]	444 [31.0]	3.45	[1.55 - 5.68]	0.002^*^	1.28	[0.48 - 3.35]	0.212
No	970 [67.8]	10 [0.7]	960 [67.1]	1			1		

AOR, Adjusted odds ratio; COR, Crude odds ratio; HBV, hepatitis B virus; n, Frequency;

* , p  <  0.05; %, Percentage

For malaria mono-infection, the bivariate analysis showed that fear of side effects, absence of signs and symptoms, forgetfulness, perceived efficacy of herbal medicine, pain, uncertainty, and fatigue due to prolonged adherence were significantly associated with malaria, as presented in **Table 7**. In the multivariate analysis, fear of side effects, absence of signs or symptoms, forgetfulness, perceived efficacy of herbal medicine, pain, uncertainty about the necessity of the interventions, and prolonged period of adherence to interventions were independently linked to the risk of malaria mono-infection. Pregnant women who emphasised non-adherence due to fear of side effects had 2.07 times the odds of being infected with malaria compared with those who adhered regardless of side effects (AOR = 2.07; 95% CI: 1.66 – 3.72). Likewise, there were 1.82 times the odds of being infected with malaria among pregnant women who did not adhere to malaria interventions (LLINs and IPTp-SP) due to the absence of signs or symptoms of malaria compared with those who adhered regardless of the absence of signs or symptoms (AOR = 1.82; 95% CI: 1.53 – 2.26). Also, the odds of malaria infection were twice as high among pregnant women who emphasised forgetfulness as a psychological barrier to adherence to interventions (AOR = 2.41; 95% CI: 1.48 – 3.90). In addition, the odds of malaria infection were two times higher among pregnant women who had a preference for herbal medicine because of its perceived efficacy as a reason for non-adherence to the interventions (AOR = 2.35; 95% CI: 1.49 – 3.68). Furthermore, pregnant women who did not adhere to the interventions due to pain had 2.52 times the odds of being infected with malaria (AOR = 2.52; 95% CI: 1.48 – 4.27). Moreover, pregnant women who expressed uncertainty about the necessity of the interventions had higher odds of being infected with malaria (AOR = 2.38; 95% CI: 1.48 – 3.84). Finally, a prolonged period of adherence as emphasised by the studied pregnant women was significantly associated with 1.71 times the odds of being infected with malaria (AOR = 1.71; 95% CI: 1.10 – 2.66).

**Table 7:** Association between psychological barriers and malaria mono-infection among pregnant women.

		Malaria status							
	Frequency	Positive	Negative	Model I			Model II		
Variable	n [%]	n [%]	n [%]	COR	[95%CI]	p-value	AOR	[95%CI]	p-value
**Fear of side effects**									
Yes	358 [25.0]	67 [4.7]	291 [20.3]	2.57	[1.82 - 3.63]	0.000^*^	2.07	[1.66 - 3.72]	0.030^*^
No	1072 [75.0]	88 [6.2]	984 [68.8]	1			1		
**Absence of signs or symptoms**									
Yes	599 [41.9]	88 [6.2]	551 [35.7]	1.96	[1.40 - 2.75]	0.000^*^	1.82	[1.53 - 2.26]	0.032^*^
No	831 [58.1]	67 [4.7]	764 [53.4]	1			1		
**Forgetfulness**									
Yes	237 [16.6]	67 [4.7]	170 [11.9]	4.94	[3.46 - 7.06]	0.000^*^	2.41	[1.48 - 3.90]	0.000^*^
No	1193 [83.4]	88 [6.2]	1105 [77.3]	1			1		
**Perceived efficacy of herbs**									
Yes	332 [23.2]	74 [5.2]	258 [18.0]	3.6	[2.55 - 5.07]	0.000^*^	2.35	[1.49 - 3.68]	0.000^*^
No	1098 [76.8]	81 [5.7]	1017 [71.1]	1			1		
**Unmet expectations of care**									
Yes	106 [7.4]	14 [1.0]	92 [6.4]	1.27	[0.70 - 2.30]	0.216	1.12	[0.64 - 1.94]	0.184
No	1324 [92.6]	141 [9.9]	1183 [82.7]	1			1		
**Pain**									
Yes	189 [13.2]	59 [4.1]	130 [9.1]	5.41	[3.73 - 7.84]	0.000^*^	2.52	[1.48 - 4.27]	0.001^*^
No	1241 [86.8]	96 [6.7]	1145 [80.1]	1			1		
**Uncertainty**									
Yes	386 [27.0]	87 [6.1]	299 [20.9]	4.17	[2.96 - 5.88]	0.000^*^	2.38	[1.48 - 3.84]	0.000^*^
No	1044 [73.0]	68 [4.8]	976 [68.3]	1			1		
**Mistrust**									
Yes	258 [18.0]	29 [2.0]	229 [16.0]	1.05	[0.68 - 1.61]	0.219	0.63	[0.37 - 1.09]	0.104
No	1172 [82.0]	126 [8.8]	1046 [73.1]	1			1		
**Distress**									
Yes	169 [11.8]	16 [1.1]	153 [10.7]	0.84	[0.48 - 1.45]	0.142	0.54	[0.28 - 1.05]	0.070
No	1261 [88.2]	139 [9.7]	1122 [78.5]	1			1		
**Prolonged period of adherence**									
Yes	460 [32.2]	76 [5.3]	384 [26.9]	2.23	[1.59 - 3.12]	0.000^*^	1.71	[1.10 - 2.66]	0.016^*^
No	970 [67.8]	79 [5.5]	891 [62.3]	1			1		

AOR, Adjusted odds ratio; COR, Crude odds ratio; n, Frequency;

* , p  <  0.05; %, Percentage

## Discussion

### Compliance coverage of HBV and malaria interventions

Given the substantial public health burden posed by infectious diseases during pregnancy in endemic regions, this study examined the coverage and adherence to key health interventions among pregnant women in the Bono East Region of Ghana, specifically focusing on hepatitis B virus (HBV) vaccination, the use of long-lasting insecticidal nets (LLINs), and Intermittent Preventive Treatment with Sulfadoxine-Pyrimethamine (IPTp-SP). These interventions are central to the World Health Organization’s (WHO) recommended strategies for significantly reducing the burden of maternal HBV and malaria [19]. To promote effective implementation, the WHO has integrated HBV vaccination into routine immunisation programs globally, particularly in regions with high endemicity [10]. Historically, HBV vaccines have been available since 1982, and by early 2011, 179 countries had included them in their national immunisation schedules, resulting in a global coverage rate of approximately 75% [10]. Coverage rates, however, vary by region, with the Americas reaching 90%, Europe 78%, Africa 76%, and Southeast Asia 52% [10]. Initially, it was assumed that HBV vaccines provided immunity for only five to seven years. However, recent studies suggest that immunity may persist for a decade or longer, potentially offering long-term or even lifelong protection after the full vaccination course [24]. Despite these global gains, this study found that HBV vaccination rates among pregnant women in the Bono East Region remained alarmingly low. This observation is consistent with previous studies that have similarly reported poor uptake of HBV vaccination among pregnant women in Ghana [25].

In a related manner, the use of Long-Lasting Insecticidal Nets (LLINs) has been identified as one of the most effective and appropriate interventions for malaria prevention [26]. These nets provide dual protection by killing mosquitoes on contact and repelling them, thus reducing the risk of mosquito bites and associated pregnancy complications [27]. Nevertheless, widespread usage remains limited. The majority of pregnant women in the study reported inconsistent or non-use of LLINs, reflecting trends seen in other malaria-endemic regions such as sub-Saharan Africa, Southeast Asia, and Latin America, despite substantial investments in this specific malaria control initiative [26].

Likewise, Intermittent Preventive Treatment with Sulfadoxine-Pyrimethamine (IPTp-SP) has gained recognition as the most effective antimalarial prophylaxis for pregnant women.. This preference stems from its favourable safety profile during pregnancy, efficacy in women of reproductive age, and suitability for single-dose administration under direct observation by healthcare workers [18,19]. Compared to chloroquine, which has traditionally been used during pregnancy, IPTp-SP is preferred due to increasing *Plasmodium* resistance to chloroquine in many endemic regions, including Ghana [28]. Both Sulphadoxine and Pyrimethamine are generally considered safe during the second and third trimesters of pregnancy [29]. Although concerns have been raised regarding potential sulphur drug-related complications such as kernicterus in premature neonates, these have not been observed in IPTp-SP studies. Indeed, most studies evaluating foetal risks from in-utero exposure to SP combinations have not reported increased incidences of spontaneous abortions or congenital anomalies [29]. While one retrospective study did report a higher risk of birth defects from antifolate drugs used in the first trimester, no such risks were identified in the second or third trimesters [30].

### Psychological determinants of non-compliance with the interventions

To extend the scope of analysis, the study also assessed the psychological determinants of non-compliance with integrated interventions and their association with HBV and malaria. As part of the determinants revealed, fear of side effects, pain, and prolonged periods of adherence were revealed in some earlier studies [31,32]. A plausible explanation for this could be that the HBV vaccines, LLINs, and IPTp-SP have been associated with several discomforts. These include headache, fever, irritability, loss of appetite, nausea, fatigue, dizziness, heat, and skin itching [33,34]. Over time, the continued use of these interventions may lead pregnant women to overestimate their effectiveness and associate their uptake with negative outcomes. This may ultimately influence adherence and could as a result increase the risk of infections, as observed in this study.

Furthermore, the study revealed that the absence of noticeable signs and symptoms along with forgetfulness were barriers to adherence, which is consistent with a previous study [35]. This could largely be attributed to inferior awareness and knowledge of the infections, their transmission, and risk factors. Both HBV and malaria may be asymptomatic, hence, many pregnant women may not perceive the urgency to follow through with medical interventions and recommended preventive guidelines when they do not feel sick, thereby compromising their adherence to interventions [36].

In addition, the perceived efficacy of alternative medicine, the perception that formal care did not meet expectations, and the lack of trust in healthcare providers were also revealed as barriers to compliance with the interventions. These also corroborate some earlier reports [32,35,37]. Given the widespread use of traditional medicine in Africa and Asia, particularly due to the accessibility, affordability, and cultural acceptance of herbal remedies [38], many pregnant women may prefer these alternativesThey often view herbal formulations as having minimal side effects and offering quicker relief. Cultural beliefs, familial influences, and personal experiences further reinforce this preference [39], which may ultimately foster mistrust in conventional healthcare systems and reduce compliance with medically recommended interventions..

Moreover, uncertainty regarding the necessity of these interventions also emerged as a notable barrier, echoing findings from previous studies [32,40]. This barrier stems from the lack of clarity or confusion regarding the necessity, benefits, and potential side effects of the interventions [32,40]. The revealed barrier could also be attributed to inadequate education or information during ANC visitation and the cultural orientation of pregnant women. This highlights the importance of effective communication strategies during ANC sessions to ensure informed decision-making.

Similarly, the study revealed distress over the death of a family member due to interventions as another barrier to adherence, which was also reported in a study to ascertain the psychosocial barriers to medication adherence in patients with type 2 diabetes [41]. The loss of a loved one may impose significant psychological strain, manifesting as grief, fear, depression, or anxiety, which in turn disrupts consistent engagement in positive health behaviours. [42]. Therefore, integrating mental health education and psychosocial support into routine ANC services could help sensitise pregnant women who have experienced such traumatic losses, and ultimately improve adherence to preventive interventions.

### Understanding non-compliance using the Health Belief Model (HBM)

Understanding the behavioural determinants of disease prevention is essential for shaping effective maternal health policies. This study employed the Health Belief Model (HBM) to explore the factors influencing pregnant women’s adherence to preventive measures against hepatitis B virus (HBV) and malaria. Drawing from existing literature, the study examined key HBM components, namely, perceived susceptibility, perceived severity, perceived benefits, perceived barriers, cues to action, and self-efficacy. The relatively low prevalence of HBV (1.8%) compared with malaria (10.8%) likely influenced women’s perceptions of susceptibility and severity. As a result, many did not view either disease as an immediate threat, particularly in the absence of symptoms. This sentiment was illustrated by statements such as, “*I rarely experience headaches, high body temperature, or joint pains*.” Consequently, the lack of perceived risk reduced the motivation to engage in preventive behaviours. In addition, emotional responses to adverse outcomes such as the reported death of a relative following HBV vaccination amplified fears and misconceptions about intervention safety, further distorting the perceived severity.

The study also identified several barriers that hindered adherence. These included fear of side effects, absence of symptoms, forgetfulness, trust in traditional herbal remedies, dissatisfaction with formal healthcare, pain, uncertainty, misplaced trust in healthcare providers, emotional distress, and fatigue from prolonged intervention regimens. These barriers closely align with the HBM framework. Although cues to action, such as health education and outreach, proved effective in raising awareness, inconsistent communication often eroded trust in healthcare providers. One participant noted, “*Some nurses here do not take the time to explain the importance of the injection*,” highlighting the critical role of provider-patient communication in reinforcing adherence. Enhancing individual capacity to act is vital for improving preventive health behaviours. Self-efficacy significantly influenced participants’ ability to act on preventive measures. A substantial proportion (27%) expressed uncertainty about the necessity of interventions, indicating low confidence in navigating healthcare services. Enhancing self-efficacy through simplified schedules, reminder systems, and supportive counselling could help improve adherence rates.

### Implications for policy and practice

The study highlights several critical policy implications that could drive meaningful change in public health efforts aimed at improving maternal and neonatal health outcomes. For policy, the finding of this study stresses the need for strengthening healthcare provider-patient communication through patient-centred approaches. This strategy could be crucial for building trust and ensuring that pregnant women understand the necessity and safety of medical interventions geared towards reducing health disparities. Moreover, health education should be targeted towards increasing awareness and dispelling misconceptions about formal maternal care. Also, culturally sensitive community engagement, involving traditional leaders and influencers, could enhance and strengthen trust in formal healthcare while countering reliance on herbal medicine. Similarly, psychosocial support programs and mental health services should be incorporated into routine ANC to help pregnant women manage distress and improve self-efficacy. To complement these efforts, the use of mobile-based reminders and community-led follow-up systems could serve as effective tools to counter forgetfulness and enhance adherence. By leveraging digital health technologies, these solutions offer timely cues to action and strengthen continuity of care in resource-constrained settings.

### Study’s strengths and limitations

As a major strength, this study used a mixed-method approach, combining quantitative data from a large and diverse sample of pregnant women (1430) with qualitative insights. This multidimensional analysis moves beyond surface-level explanations to uncover deeper psychological and emotional influences that shape health-seeking behaviour. In addition, this study extended the application of the Health Belief Model (HBM) to demonstrate how perceptions of susceptibility, severity, benefits, and barriers influence non-compliance with interventions. In addition, the large sample size employed enhances the generalizability of the findings. Finally, the study used thematic analysis to provide depth, texture, and contextual clarity into the experiences of pregnant women regarding non-compliance that cannot be conveyed through numerical summaries alone. Despite its strengths, the study has several limitations. Firstly, the cross-sectional design limits the ability to infer causality between identified barriers and adherence to the interventions. In addition, while the study’s large sample size enhances generalizability within the Bono East Region, the findings may not be fully applicable to other regions with different sociocultural contexts. Finally, the qualitative data, although rich and insightful, is inherently subjective and may be influenced by the participants’ prior conception.

## Conclusion

Unlike previous studies that have primarily examined epidemiological trends and coverage rates, this current study uniquely integrates psychological barriers with actual health outcomes to establish direct associations between these barriers and viral hepatitis B (HBV) and malaria infections. By adopting this approach, it bridges the gap between behavioural health insights and infectious disease prevention, thereby offering a more holistic understanding of the factors driving non-compliance key interventions [HBV vaccination, long-lasting insecticidal nets (LLINs), and intermittent preventive treatment with sulfadoxine-pyrimethamine (IPTp-SP)]. Notwithstanding global efforts to integrate these interventions into routine maternal healthcare, adherence remains suboptimal due to psychological barriers such as fear of side effects, prolonged adherence fatigue, absence of symptoms, forgetfulness, perceived efficacy of herbal medicine, unmet expectations of formal care, pain, uncertainty, and emotional distress from family loss. These findings highlight the urgent need for actionable public health strategies that go beyond clinical service provision. Some of these strategies include psychosocial and mental health care, enhanced patient-provider communication, culturally tailored health education, and targeted behavioural interventions. Addressing these psychological barriers is essential not only for improving intervention uptake but also for reducing the maternal and foetal burden of HBV and malaria. Looking ahead, future research should explore longitudinal trends in adherence and evaluate the effectiveness of intervention-based strategies such as digital health tools, behavioural nudges, and tailored education campaigns to enhance compliance. Such efforts are essential for improving maternal and child health outcomes in high-burden, low-resource settings.

## Supporting information

S1 Text Questionnaire. (DOCX)

S2 Text Clinical Data Collection Tool. (DOCX)

S3 Text Focus Group Discussion Guide. (DOCX)

S4 Text In-depth Interview Guide. (DOCX)

S1 Data Dataset. (XLSX)
