# Supplementary material for: From need to neglect: Exploring psychological barriers to preventive interventions in pregnancy
Source: PLOS Glob Public Health. 2025 Jun 24;5(6):e0004826. doi: 10.1371/journal.pgph.0004826 (PMC12186947; doi:10.1371/journal.pgph.0004826)
Supplement: S1 Text — (DOCX) [file pgph.0004826.s001.docx]

**AKENTEN APPIAH MENKA UNIVERSITY OF SKILL TRAINING AND ENTREPRENEURIAL DEVELOPMENT**

**DEPARTMENT OF PUBLIC HEALTH EDUCATION**

**SURVEY ON PREVALENCE OF HBV AND MALARIA AMONG PREGNANT WOMEN**

This questionnaire is part of a survey for MPhil work in Public Health being undertaken by Dennis Bardoe a student at the Department of Public Health Education, Akenten Appiah Menka University of Skill Training and Entrepreneurial Development. This research aims to determine the prevalence of HBV and malaria co-infection among pregnant women in the Bono East Region of Ghana. The study is based on a selected sample, so your participation is critical. Therefore, I would be most grateful if you could spare a few minutes to complete this questionnaire.

The results of this research will help fill a critical knowledge gap by determining the prevalence of HBV and malaria mono- or co-infection, risk factors, socio-demographic predictors of HBV and malaria co-infection, and barriers to adherence to HBV and malaria preventive guidelines or interventions among pregnant women in Ghana’s Bono East Region. The findings of this study will also assist stakeholders such as the Ministry of Health, Ghana Health Service, and other healthcare-related organizations in developing practical measures regarding prevention, screening, monitoring, and treatment to improve maternal and neonatal health

You are assured that any information you provide will be treated with strict confidentiality. Your anonymity is also guaranteed. Your participation is voluntary. You were selected as a participant because you agreed to participate. It will take about 45 minutes of your time. There are no anticipated risks to your participation.

# INTERVIEW INFORMATION

DATE OF INTERVIEW |__|__| Day |__|__| Month |__|__||__|__| Year

TIME STARTED |__|__| Hour |__|__| Minutes

TIME ENDED |__|__| Hour |__|__| Minutes

RESULT ^*^ |__|

INTERVIEWER NAME ______________________________________

RESIDENCE MUNICIPALITY ___________________________________­­___

RESIDENCE COMMUNITY ______________________________________

ENROLMENT CODE ______________________________________

GROUP CODE ______________________________________

RESPONDENT CELLPHONE NUMBER |__|__||__|__||__|__||__|__||__|__|

*RESULT CODES:

1=COMPLETED 4=REFUSED 5=OTHER (SPECIFY) 2=PARTLY COMPLETED 3=POSTPONED

*AGE GROUP

| 1 = 15 - 20 | 2 = 21 - 25 | 3 = 26 - 30 | 4 = 31 - 35 | 5 = 36 - 40 |
| --- | --- | --- | --- | --- |
| 6 = 41 - 45 | 7 = 46 - 50 | 8 = 51 - 55 | 9 = 56 - 60 | 10 = 61 - 64 |

On the assessment of the knowledge, a score of 0-3 (Not very likely or Strongly disagree) will be regarded as extremely low, 4-6 (Somewhat likely or Disagree) will be regarded as low, 7-9 (Very likely or Agree) will be regarded as moderate, and 10-12 (Extremely likely or Strongly agree) will be regarded as high. For the assessment of attitude, a score of 0-2 (No) will be regarded as bad and a score of 3-4 (Yes) will be regarded as good. Composite scores were obtained from individual scorings and put together or categorized to ascertain the percentage of the population with low, moderate, or high knowledge and poor or good attitude towards the HBV infection.

# QUESTIONNAIRE

## SECTION A: SOCIODEMOGRAPHIC CHARACTERISTICS

| I would like to start by asking you a few questions about yourself. | | | |
| --- | --- | --- | --- |
| No. | Question | Response options | Skip |
|  | Age | 1. Between 18 and 25 [ ] 2. Between 26 and 30 [ ] 3. Between 31 and 40 [ ] 4. Between 41 and 49 [ ] 5. Between 51 and 60 [ ] |  |
|  | Marital Status: | 1. Never married [ ] 2. Married [ ] 3. Cohabitation [ ] |  |
|  | How long have you been married/living together? | 1. Between 1 and 11 months [ ] 2. Between 1 and 10 years [ ] 3. Between 11 and 20 years [ ] 4. Between 21 and 30 years [ ] |  |
|  | Education | 1. No formal education [ ] 2. Primary [ ] 3. JHS/Middle School [ ] 4. SHS [ ] 5. Tertiary [ ] 6. Technical Vocational Education [ ] |  |
|  | Religious Affiliation | 1. Islam [ ] 2. Christianity [ ] 3. African Traditional Religion [ ] 4. Others [ ] |  |
|  | Occupational status | 1. Employed [ ] 2. Unemployed [ ] |  |
|  | On the average, how much do you earn from your work monthly? | 1. Between Ghȼ 100 and Ghȼ 500 [ ] 2. Between Ghȼ 600 and Ghȼ 1000 [ ] 3. Between Ghȼ 1100 and Ghȼ 2000 [ ] 4. Between Ghȼ 2100 and Ghȼ 3000 [ ] 5. Between Ghȼ 3100 and Ghȼ 4000 [ ] |  |
|  | Which of the following best describes the household structure? | 1. Female Centered (No husband/ male partner in household, may include relatives, children, friends) [ ] 2. Male-Centered (No wife/ female partner in household, may include relatives, children, friends) [ ] 3. Nuclear (Husband/ male partner and wife/ female partner with or without children) [ ] 4. Extended (Husband/ male partner and wife/ female partner and children and relatives) [ ] 5. Polygamous (husband with more than one wife) [ ] |  |
|  | Which one of the following housing types best describes  the type of dwelling of your household? | 1. Self-contain house [ ] 2. Compound house [ ] |  |
|  | Which one of the following housing categories best describes  the type of dwelling of your household? | 1. Mud with thatch [ ] 2. Mud with iron sheets [ ] 3. Bricks with thatch [ ] 4. Bricks with iron sheets [ ] 5. Blocks with thatch [ ] 6. Blocks with iron sheets [ ] |  |
|  | What is the main occupation of your household? | 1. Farming [ ] 2. Fishing [ ] 3. Trading/business [ ] 4. Civil service [ ] 5. Unemployed [ ] 6. Preferred not to answer [ ] |  |
|  | How many people in total live in your household? | 1. Between 1 and 5 [ ] 2. Between 6 and 10 [ ] 3. Between 11 and 15 [ ] 4. Between 16 and 20 [ ] 5. Between 21 and 25 [ ] 6. Between 26 and 30 [ ] |  |

## SECTION B: BARRIERS TO HBV AND MALARIA INTERVENTIONS

### PSYCHOLOGICAL BARRIERS

| **PSYCHOLOGICAL BARRIERS**  Here are some questions about why people fail to adhere to interventions. I will read you a statement. Please answer which of the options is most closely suited to you. | | | |
| --- | --- | --- | --- |
| No. | Question | Response options | Skip |
|  | Concern about the effect of the intervention | 1. Yes [ ] 2. No [ ] 3. Don’t know [ ] 4. Prefer not to say [ ] |  |
|  | I am afraid of the interventions and/or the side effects (Fear of side effects) (Fear) | 1. Yes [ ] 2. No [ ] 3. Don’t know [ ] 4. Prefer not to say [ ] |  |
|  | Generally, I often feel downcast and sometimes discouraged and depressed | 1. Yes [ ] 2. No [ ] 3. Don’t know [ ] 4. Prefer not to say [ ] |  |
|  | I often forget things during my daily routine (Forgetfulness) | 1. Yes [ ] 2. No [ ] 3. Don’t know [ ] 4. Prefer not to say [ ] |  |
|  | Feel sad to adhere intervention | 1. Yes [ ] 2. No [ ] 3. Don’t know [ ] 4. Prefer not to say [ ] |  |
|  | Feel nervous about adhering to intervention | 1. Yes [ ] 2. No [ ] 3. Don’t know [ ] 4. Prefer not to say [ ] |  |
|  | Feel pain to intervention? | 1. Yes [ ] 2. No [ ] 3. Don’t know [ ] 4. Prefer not to say [ ] |  |
|  | Sometimes I am unsure whether the interventions are indeed necessary (uncertainty) | 1. Yes [ ] 2. No [ ] 3. Don’t know [ ] 4. Prefer not to say [ ] |  |
|  | Concern due to the lack of trust in health service providers | 1. Yes [ ] 2. No [ ] 3. Don’t know [ ] 4. Prefer not to say [ ] |  |
|  | Distress about the death of a family member (sister, brother) | 1. Yes [ ] 2. No [ ] 3. Don’t know [ ] 4. Prefer not to say [ ] |  |
|  | Getting tired of the prolonged period of treatment | 1. Yes [ ] 2. No [ ] 3. Don’t know [ ] 4. Prefer not to say [ ] |  |
