# Supplementary material for: From need to neglect: Exploring psychological barriers to preventive interventions in pregnancy
Source: PLOS Glob Public Health. 2025 Jun 24;5(6):e0004826. doi: 10.1371/journal.pgph.0004826 (PMC12186947; doi:10.1371/journal.pgph.0004826)
Supplement: S2 Text — (DOCX) [file pgph.0004826.s002.docx]

**AKENTEN APPIAH MENKA UNIVERSITY OF SKILL TRAINING AND ENTREPRENEURIAL DEVELOPMENT**

**DEPARTMENT OF PUBLIC HEALTH EDUCATION**

**SURVEY ON PREVALENCE OF HBV AND MALARIA AMONG PREGNANT WOMEN**

This questionnaire is part of a survey for MPhil work in Public Health being undertaken by Dennis Bardoe a student at the Department of Public Health Education, Akenten Appiah Menka University of Skill Training and Entrepreneurial Development. This research aims to determine the prevalence of HBV and malaria co-infection among pregnant women in the Bono East Region of Ghana. The study is based on a selected sample, so your participation is critical. Therefore, I would be most grateful if you could spare a few minutes to complete this questionnaire.

The results of this research will help fill a critical knowledge gap by determining the prevalence of HBV and malaria mono- or co-infection, risk factors, socio-demographic predictors of HBV and malaria co-infection, and barriers to adherence to HBV and malaria preventive guidelines or interventions among pregnant women in Ghana’s Bono East Region. The findings of this study will also assist stakeholders such as the Ministry of Health, Ghana Health Service, and other healthcare-related organizations in developing practical measures regarding prevention, screening, monitoring, and treatment to improve maternal and neonatal health

You are assured that any information you provide will be treated with strict confidentiality. Your anonymity is also guaranteed. Your participation is voluntary. You were selected as a participant because you agreed to participate. It will take about 45 minutes of your time. There are no anticipated risks to your participation.

# INTERVIEW INFORMATION

DATE OF INTERVIEW |__|__| Day |__|__| Month |__|__||__|__| Year

TIME STARTED |__|__| Hour |__|__| Minutes

TIME ENDED |__|__| Hour |__|__| Minutes

RESULT ^*^ |__|

INTERVIEWER NAME ______________________________________

RESIDENCE MUNICIPALITY ___________________________________­­___

RESIDENCE COMMUNITY ______________________________________

ENROLMENT CODE ______________________________________

GROUP CODE ______________________________________

*RESULT CODES:

1=COMPLETED 4=REFUSED 5=OTHER (SPECIFY) 2=PARTLY COMPLETED 3=POSTPONED

# CLINICAL RECORDS

| **Clinical Record** | **Operational definition** | **Result** | | | | | | | | |
| --- | --- | --- | --- | --- | --- | --- | --- | --- | --- | --- |
| Gravidity | Number of pregnancies a participant has had including the current |  | | | | | | | | |
| Parity | The number of children ever born to a woman |  | | | | | | | | |
| ANC Visits | Number of times a participant had visited the antenatal care clinic during the current pregnancy |  | | | | | | | | |
| Gestation | The trimester in which a participant attended the first ANC clinic |  | | | | | | | | |
| Blood Group | Participant’s blood group |  | | | | | | | | |
| Haemoglobin (HB) level | Participant's HB level | 1ST | 2ND | | 3RD | | 4TH | 5TH | | 6TH |
|  |  |  |  | |  | |  |  | |  |
|  |  | AVERAGE HB | | | | |  | | | |
| Anaemia | Participant's anaemia status |  | | | | | | | | |
| Sickling | Sickling status of a participant |  | | | | | | | | |
| Glucose-6-phosphate Dehydrogenase (G6PD) | G6PD status |  | | | | | | | | |
| Syphilis | Participant's Syphilis status |  | | | | | | | | |
| HIV | Participant's HIV status |  | | | | | | | | |
| HBV vaccination | HBV vaccination coverage | Yes | | | | No | | | | |
| LLINs usage |  | Yes | | | | No | | | | |
| IPTp-SP uptake |  | ≤1 | | 2 | | 3 | | | ≥4 | |
| ANC - Antenatal Care, HB - Haemoglobin, HIV – Human Immune Virus, HBV - Hepatitis B Virus, G6PD-Glucose-6-phosphate dehydrogenase, LLINS-, long-lasting insecticidal nets, IPTp-SP-intermittent preventive treatment in pregnancy with sulfadoxine-pyrimethamine | | | | | | | | | | |
