# Supplementary material for: From need to neglect: Exploring psychological barriers to preventive interventions in pregnancy
Source: PLOS Glob Public Health. 2025 Jun 24;5(6):e0004826. doi: 10.1371/journal.pgph.0004826 (PMC12186947; doi:10.1371/journal.pgph.0004826)
Supplement: S3 Text — (DOCX) [file pgph.0004826.s003.docx]

**AKENTEN APPIAH MENKA UNIVERSITY OF SKILL TRAINING AND ENTREPRENEURIAL DEVELOPMENT**

**DEPARTMENT OF PUBLIC HEALTH EDUCATION**

**SURVEY ON PREVALENCE OF HBV AND MALARIA AMONG PREGNANT WOMEN**

This questionnaire is part of a survey for MPhil work in Public Health being undertaken by Dennis Bardoe a student at the Department of Public Health Education, Akenten Appiah Menka University of Skill Training and Entrepreneurial Development. This research aims to determine the prevalence of HBV and malaria co-infection among pregnant women in the Bono East Region of Ghana. The study is based on a selected sample, so your participation is critical. Therefore, I would be most grateful if you could spare a few minutes to complete this questionnaire.

The results of this research will help fill a critical knowledge gap by determining the prevalence of HBV and malaria mono- or co-infection, risk factors, socio-demographic predictors of HBV and malaria co-infection, and barriers to adherence to HBV and malaria preventive guidelines or interventions among pregnant women in Ghana’s Bono East Region. The findings of this study will also assist stakeholders such as the Ministry of Health, Ghana Health Service, and other healthcare-related organizations in developing practical measures regarding prevention, screening, monitoring, and treatment to improve maternal and neonatal health

You are assured that any information you provide will be treated with strict confidentiality. Your anonymity is also guaranteed. Your participation is voluntary. You were selected as a participant because you agreed to participate. It will take about 45 minutes of your time. There are no anticipated risks to your participation.

# INTERVIEW INFORMATION

DATE OF INTERVIEW |__|__| Day |__|__| Month |__|__||__|__| Year

TIME STARTED |__|__| Hour |__|__| Minutes

TIME ENDED |__|__| Hour |__|__| Minutes

RESULT ^*^ |__|

INTERVIEWER NAME ______________________________________

RESIDENCE MUNICIPALITY ___________________________________­­___

RESIDENCE COMMUNITY ______________________________________

ENROLMENT CODE ______________________________________

GROUP CODE ______________________________________

*RESULT CODES:

1=COMPLETED 4=REFUSED 5=OTHER (SPECIFY) 2=PARTLY COMPLETED 3=POSTPONED

# FOCUS GROUP DISCUSSION

INTRODUCTION

*Greetings and thank you for allowing us to have this discussion with you. We are with Akenten Appiah Menka University of Skill Training and Entrepreneurial Development (AAMUSTED), Mampong. My name is …….. and these are my colleagues…………… (Let them introduce themselves). We are conducting several meetings with people like yourself to find out your views about the risk factors of HBV and malaria among pregnant women, interventions, and barriers to adherence to intervention.*

*Your opinions are very important and they will help in the formulation of comprehensive guidelines to improve maternal and neonatal health. There are no right or wrong answers. Your contribution is very valuable. Whatever you say will be confidential so feel at ease to express your opinions.*

| PSYCHOLOGICAL BARRIERS TO COMPLIANCE WITH INTERVENTIONS | | |
| --- | --- | --- |
|  | Question | Response |
|  | Do you sleep in an Insecticide Treated Mosquito Net? |  |
|  | Have you ever been concerned about side effects from HBV vaccination, LLINs, or IPTp-SP? |  |
|  | Can you describe any specific side affects you or someone you know experienced? |  |
|  | How do these concerns influence your decision to adhere to these interventions? |  |
|  | How does the absence of symptoms of HBV or malaria affect your decision to take preventive measures? |  |
|  | Do you think it is necessary to take medication or vaccination when you feel healthy? Why or why not? |  |
|  | How do your daily responsibilities impact your ability to remember taking your medication or using LLINs? |  |
|  | What strategies, if any, do you use to help you remember? |  |
|  | What are your thoughts on traditional herbal medicine compared to HBV vaccination, LLINs, and IPTp-SP? |  |
|  | Have you ever used herbal medicine for prevention or treatment during pregnancy? Why? |  |
|  | How do you feel about the healthcare services you have received during your pregnancy? |  |
|  | Have you had any negative experiences with health service providers? |  |
|  | Can you share your experiences with pain or discomfort from any of these interventions? |  |
|  | How does this affect your willingness to continue with the intervention? |  |
|  | How important do you think HBV vaccination, LLINs, and IPTp-SP are for your health and your baby’s health? |  |
|  | Are there any doubts or questions you have about their necessity? |  |
|  | What level of trust do you have in the healthcare workers who provide these interventions? |  |
|  | What could be done to improve your trust in them? |  |
|  | How has losing a family member, if applicable, impacted your adherence to health recommendations during pregnancy? |  |
|  | Do you feel this distress has affected your ability to focus on your own health? |  |
|  | How do you feel about the long-term commitment required for these interventions? |  |
|  | What challenges do you face in maintaining adherence over time? |  |
|  | In your opinion, what are some of the recommendations to be implemented to curtail these barriers that influence pregnant women not to adhere to the interventions provided to prevent the transmission of HBV and Malaria? |  |
