# Supplementary material for: From need to neglect: Exploring psychological barriers to preventive interventions in pregnancy
Source: PLOS Glob Public Health. 2025 Jun 24;5(6):e0004826. doi: 10.1371/journal.pgph.0004826 (PMC12186947; doi:10.1371/journal.pgph.0004826)
Supplement: S4 Text — (DOCX) [file pgph.0004826.s004.docx]

# INTERVIEW INFORMATION

DATE OF INTERVIEW |__|__| Day |__|__| Month |__|__||__|__| Year

TIME STARTED |__|__| Hour |__|__| Minutes

TIME ENDED |__|__| Hour |__|__| Minutes

RESULT ^*^ |__|

INTERVIEWER NAME ______________________________________

RESIDENCE MUNICIPALITY ___________________________________­­___

RESIDENCE COMMUNITY ______________________________________

ENROLMENT CODE ______________________________________

GROUP CODE ______________________________________

*RESULT CODES:

1=COMPLETED 4=REFUSED 5=OTHER (SPECIFY) 2=PARTLY COMPLETED 3=POSTPONED

# IN-DEPTH INTERVIEW WITH KEY HEALTH WORKERS

INTRODUCTION

*Greetings and thank you for allowing us to have this discussion with you. We are with Akenten Appiah Menka University of Skill Training and Entrepreneurial Development (AAMUSTED), Mampong. My name is ……... and these are my colleagues…………… (Let them introduce themselves). We are conducting several meetings with people like yourself to find out your views about the risk factors of HBV and malaria among pregnant women, interventions, and barriers to adherence to intervention.*

*Your opinions are very important and they will help in the formulation of comprehensive guidelines to improve maternal and neonatal health. There are no right or wrong answers. Your contribution is very valuable. Whatever you say will be confidential so feel at ease to express your opinions.*

BACKGROUND INFORMATION

Name Respondent_______________________________________________________________________

Gender_______________________________________________________________________________

Designation_____________________________________________________________________________

Number of years working with the facility____________________________________________________

Municipality/District______________________________________________________________________

Name of Health Facility_____________________________________________________________________

|  | Question | Response |
| --- | --- | --- |
|  | What would you say your health facility is? | 1. CHPS 2. Sub-District Health Facility 3. Clinic 4. Hospital |
|  | Besides this community, do you serve other communities? | 1. Yes 2. No |
|  | What do you think are the most common diseases in pregnant women in this municipality/district you serve and why? |  |
|  | What about HBV and Malaria (If not mentioned) |  |
|  | What do you think are the severity of these two diseases, especially during pregnancy? |  |
|  | Has there been any birth complications suffered by a pregnant woman as a result of these infections? |  |
|  | In your opinion, what are some of the risk factors associated with the transmission of these infections? |  |
|  | Are you involved in delivering an intervention, activity or strategy that aims to improve the uptake of HBV and Malaria interventions? |  |
|  | Does your facility provide education/sensitization on HBV and Malaria? |  |
|  | When do you start your education on HBV and Malaria? |  |
|  | Does your service promote and/or offer testing for HBV and Malaria? |  |
|  | If yes, where do you get your test kits from? |  |
|  | Are the test kits always available? |  |
|  | If No, why are your clients not tested? |  |
|  | Do you have records on HBV and Malaria testing? |  |
|  | What are the challenges with the HBV and Malaria testing? |  |
|  | Do you vaccinate your clients (pregnant women)? |  |
|  | If yes, where do you get vaccines from? |  |
|  | How do you keep the vaccines? |  |
|  | If No, how do they (pregnant women) get vaccinated? |  |
|  | In your opinion, what are some of the psychological barriers that influence non-compliance pregnant women not to adhere to the interventions you provide |  |
